# Self-care in stroke rehabilitation: a qualitative study

**DOI:** 10.1590/0034-7167-2025-0332

**Published:** 2026-07-17

**Authors:** Victor Nhime Nungulo, Mauer Gonçalves, Maria Adriana Henriques, Cristina Lavareda Baixinho

**Affiliations:** I Universidade José Eduardo dos Santos. Huambo, Angola; II Universidade de Lisboa. Lisboa, Portugal; III Universidade Agostinho Neto. Luanda, Angola; IV Center for Innovative Care and Health Technology. Leiria, Portugal

**Keywords:** Self Care, Caregivers, Health Personnel, Qualitative Research, Stroke Rehabilitation., Autocuidado, Cuidadores, Personal de Salud, Investigación Cualitativa, Rehabilitación de Accidente Cerebrovascular

## Abstract

**Objectives:**

to analyze caregivers’ and health professionals’ perceptions of interventions that promote self-care in stroke survivors.

**Methods:**

qualitative study within an interpretivist paradigm; we conducted nine interviews with family caregivers and three focus groups with health professionals. Data were collected in Angola (December 2024-January 2025), audio-recorded, transcribed, and analyzed using content analysis in webQDA^®^.

**Results:**

central concerns include planning and continuity of individualized rehabilitation programs to ensure ongoing support, communication, and access to resources; training stroke survivors and their families for self-care management through information and education, prevention of self-neglect, and support for therapeutic regimen management; and training family caregivers to promote self-care and transition into the caregiver role.

**Final Consideration:**

the findings clarify caregivers’ and health professionals’ perceptions of how self care is promoted among stroke survivors and yield recommendations for clinical practice and research.

## RESUMEN

**Objetivos:**

analizar las percepciones de cuidadores familiares y profesionales de la salud sobre las intervenciones que promueven el autocuidado en personas supervivientes de ictus.

**Métodos:**

estudio cualitativo en el paradigma interpretativo; nueve entrevistas a cuidadores familiares y tres grupos focales con profesionales; datos recogidos en Angola (diciembre 2024-enero 2025), con grabación y transcripción para análisis de contenido en WebQDA^®^.

**Resultados:**

preocupaciones centrales: planificación y continuidad de programas individuales de rehabilitación para garantizar apoyo, comunicación y recursos; capacitación para la gestión del autocuidado (información, educación, prevención de la negligencia y apoyo en la gestión terapéutica); y capacitación del cuidador familiar para promover el autocuidado y la transición al rol de cuidador.

**Conclusiones:**

los hallazgos aclaran percepciones sobre la promoción del autocuidado en personas supervivientes de ictus y generan recomendaciones para la práctica clínica y la investigación.

## INTRODUCTION

Stroke is one of the leading causes of disability and death, impairing functional ability and quality of life not only for stroke survivors but also for caregivers and family members^(1-3)^. After a stroke, survivors may have temporary or permanent disability that affects self-care, making them dependent on a caregiver or health professional to perform activities of daily living. Impairments in self-care are a concern because they affect the progression of functional ability in the months following stroke onset^(4,5)^.

A systematic review showed that these individuals have multiple needs stemming from physical impairments, functional disability, emotional lability, cognitive impairment, fatigue, and chronic pain, as well as changes in participation in family and social life caused primarily by limitations in mobility and communication^(1-5)^. There are also unmet needs related to difficulties accessing health care and ensuring continuity of care across hospital, rehabilitation, and home settings^(5)^.

Post-stroke sequelae, combined with health systems’ challenges in ensuring continuity of care and long-term support, limit self-care and the adoption of behaviors that support healthy living, emotional well-being, and participation in social life^(4-6)^.

International studies report that several interventions can promote self-care among stroke survivors, enabling engagement in activities of daily living and other activities that preserve biopsychosocial well-being^(7,8)^. There is growing research interest in psychoeducational interventions, which have demonstrated effectiveness in enabling stroke survivors and their caregivers to become independent in self-care and to manage their health condition^(1-3,8,9)^. Such actions facilitate community reintegration^(9,10)^ and build caregivers’ competence to support, without replacing, self-care, thereby strengthening health literacy and the ability to mobilize community resources to meet the needs of survivors and families^(5,11)^. In addition, these interventions reduce the economic burden on health systems associated with treating sequelae, complications, and readmissions^(12)^.

The main psychoeducational interventions implemented with stroke survivors and their families include provision of information; caregiver education, including training in daily care and physical rehabilitation; psychological support through counseling and supportive services, fostering positive emotional responses to daily stress; emotional support through support groups, active listening, or psychotherapy; assistance with priority setting and problem solving; behavioral and emotion regulation to support behavior change and improve health outcomes; and social support, including the identification of health and community services, how to access them, and strategies to obtain help from family and friends^(1,2,13)^.

Evidence indicates that these interventions are most effective when initiated during hospitalization as part of discharge planning^(7,13)^. Home care is essential to meet survivors’ needs and reduce family burden during the transition into the caregiver role; planning should, therefore, begin during hospitalization, before discharge^(13)^. The literature indicates that psychoeducational programs are more effective when they have a minimum duration of three months and include caregiver training^(1,2,13)^. The authors recommend that nurses lead these programs, oversee their implementation, and evaluate their effectiveness^(13)^.

The scarcity of studies on this topic in Africa, particularly in Angola, warrants the present study.

## OBJECTIVES

To analyze caregivers’ and health professionals’ perceptions of interventions that promote self-care in stroke survivors.

## METHODS

### Ethical aspects

The study was conducted in accordance with national and international ethical guidelines and was approved by the ethics committee of the Princess Diana Center for Physical Medicine and Rehabilitation in Huambo, Angola. Family caregivers and health professionals signed the Informed Consent Form, authorizing participation and audio/video recording, and were informed that they could withdraw at any time. Ethical aspects related to pseudonymization and confidentiality were respected. The content of the focus groups (FGs) and interviews (E) was transcribed and stored in a cloud-based qualitative data analysis software with restricted access via username and password. To preserve anonymity and confidentiality, findings were presented with alphanumeric codes: focus groups as FG1, FG2, FG3; health professionals as HCP1, HCP2 …, HCPn; and family caregivers as FC1, FC2 …, FCn.

### Study design

The research question was: “What are caregivers’ and health professionals’ perceptions of interventions that promote self-care in stroke survivors?”. To address it, we chose a study within the interpretivist paradigm. Qualitative studies allow in-depth exploration of complex phenomena and understanding of experiences in specific contexts, from participants’ perspectives, to inform solutions to intricate real-world issues^(14)^. Because the literature review revealed a scarcity of studies on this topic in Angola, we selected semi-structured interviews and focus groups as methods^(15-17)^.

Focus groups foster understanding, description, and analysis of reality through the dynamics of social relationships; they encompass the universe of meanings, motives, aspirations, beliefs, perceptions, values, attitudes, opinions, and interpretations regarding how people live, feel, and think; and they enable group interaction based on the researcher’s active role in facilitating discussion for data collection^(15)^.

The FGs were planned according to Krueger and Casey’s guidelines, following a protocol that included planning, preparation, moderation, data analysis, and dissemination of findings^(15)^.

First, interviews were conducted with caregivers; next, FGs were held with health professionals. Finally, data triangulation was performed by combining data from different sources^(16)^. This procedure led to a broader understanding of reality and revealed additional facets of the phenomenon under investigation^(17)^.

In preparing this article, we followed the COREQ (Consolidated criteria for Reporting Qualitative Research) recommendations^(18)^.

### Study setting

The participants in this study were family caregivers and health professionals who provided care to people who had experienced a stroke and were undergoing rehabilitation at the Princess Diana Center for Physical Medicine and Rehabilitation, Huambo, Angola. The center also serves individuals living at home who attend the service to continue their rehabilitation program, with or without inpatient admission. Therefore, continuity of care at home is the family’s responsibility.

Nine interviews with family caregivers and three focus groups with health professionals were conducted, using semi-structured guides tailored to each group. For the focus groups, a homogeneous sample was selected to keep the discussion focused on the topic and deepen the findings. Participants took part in only one data-collection modality, according to predefined criteria.

### Data source

Data were collected between December 2024 and January 2025 at the Princess Diana Center for Physical Medicine and Rehabilitation, Huambo, Angola.

After authorization from the center’s administration, potential participants were identified. The researcher responsible for the study made initial contact with identified professionals and family caregivers to verify eligibility.

A semi-structured interview guide was developed. The central question was: “How is self care promoted in stroke survivors?”. From this, more specific questions were developed.

The FGs were moderated by the same moderator, who also conducted the interviews, with support from a co-moderator who took notes on the discussion^(15)^. The FGs were conducted in person, and participant consent was obtained before the sessions, which were audioand video-recorded. The average duration of each FG was 90 minutes, and interviews lasted 45 minutes.

### Data analysis

After transcription of the interviews and FGs, data analysis followed recommendations for qualitative data analysis^(17)^. Data were coded and assigned to corresponding categories based on the questions listed in the interview guide as well as those that emerged during the interviews and FGs. Data were stored and retrieved after transcription. Similar textual data were grouped into the same category, enabling an organized, systematic analysis. The analysis was performed using qualitative data analysis software (webQDA^®^).

The researcher who transcribed the material performed the coding, and the other team members validated it. During categorization, we considered representativeness, data saturation, homogeneity, relevance, and comprehensiveness.

Interviews and FGs were analyzed separately. Subsequently, triangulation of results from the two data sources was performed by comparing codes to identify similarities and differences across categories and their subcategories^(16,17)^.

## RESULTS

Three FGs were conducted with health professionals (FG1, n = 12; FG2, n = 7; FG3, n = 8) and nine interviews with family caregivers (n = 9). Of the nine participating family caregivers, six were men and three were women; the mean age was 34.5 years (±9.55; range, 28-54).

The 27 health professionals represented the following professional categories: nurse (n = 12), physical therapist (n = 10), social worker (n = 2), psychologist (n = 1), physician (n = 1), and respiratory therapist (n = 1). Eighteen were women and nine were men, with ages ranging from 29 to 52 years [39.1 (±6.39)]. The mean professional experience was 10 years.

Analysis of the FGs and interviews yielded 208 coding units, distributed across the following categories: 1) Planning and continuity of individualized rehabilitation programs, with the subcategories: support, communication, and resources for self-care; 2) Training stroke survivors and their families for self-care management, with the subcategories: professional interventions that promote self-care (information for self-care, education, prevention of self-neglect, caregiver training, and support for therapeutic regimen management) and accountability; 3) Training family caregivers, with the subcategories: skills training, support to promote independence, and transition into the caregiver role (Chart 1).

**Chart 1:** Representation of categories and subcategories that emerged from the analysis of the findings, Huambo, Angola, 2025

Categories	Subcategories
Planning and continuity of individualized rehabilitation programs	Support Communication Resources for self-care
Training stroke survivors and their families for self-care management	Professional interventions that promote self-care Accountability
Training family caregivers	Skills training Support to promote independence Transition into the caregiver role

With regard to the category Planning and continuity of individualized rehabilitation programs, participants emphasized the importance of supporting the survivor-family dyad to ensure continuity of care at home.

Family caregivers’ accounts focused on support needs because, after a stroke:

*The biggest challenge was that it was a new situation and I had to take care of his basic needs.* (FC5)

They noted that in the hospital, there is limited time or opportunity to receive explanations about what to do regarding patients’ limitations related to hemiparesis, hemiplegia, and mobility and communication difficulties. Therefore, the support provided by professionals at the rehabilitation center is important not only to help them understand the sequelae and the implications of the condition for self-care but also to learn procedures to ensure continuity of care at home, since these survivors travel to the center for rehabilitation but are not admitted. A family caregiver explained this situation:

[…] *at the first appointment, they always give guidance on how to provide care and also teach her how to perform self-care. They told me not to be anxious, to help her with bathing and feeding, and to teach her personal hygiene* […] *so that she can become able to do it on her own.* (FC8)

Professionals at the rehabilitation center were aware that survivors and families were unprepared for this new reality of dependence-these are adults who, until then, had been independent in self-care:

*Treating patients with post-stroke sequelae is challenging because the problems begin within the family setting, with the economic situation and the difficulties of transporting the patient from home to the center. One of the major challenges is that the patient and family often do not accept the condition* […]*. One of the biggest problems is acceptance and the care required for the stroke survivor, especially by family members, to ensure continuity of care at home.* (FG1, HCP6)

Families’ lack of preparation to deal with the situation, survivors’ functional changes, difficulties accessing the rehabilitation center due to transportation, and even a lack of awareness of the need to maintain a rehabilitation program lead professionals to opt for home visits to support the dyad:

*We often made home visits to assess whether the patient is able to perform activities of daily living and to teach self-care* […] *psychological support is also critical.* (FG2, HCP5)

[…] *regarding follow-up of our patients at home, we first need to see the conditions in which the patient lives and guide adaptations to the residence. For example, for those who use a wheelchair, the house is often not prepared for that purpose, which can make things difficult. Knowing these conditions prevents the patient from having an accident at home. As my colleague mentioned earlier, supporting caregivers is also important because they think treatment takes only a few days* […] *no! Stroke care involves ongoing follow-up, and engaging the family is essential. The family needs to know that their patient has this problem, but it is not the end of life. Teaching the patient to accept this condition helps motivate recovery and a return to activities of daily living, although, at times, irreversible sequelae remain.* (FG2, HCP7)

All interviewees agreed that communication is essential and central to training and continuity of care, but they reported difficulties in verbal and written communication both across and within teams, as well as in interactions with patients and families.

Communication among services and professionals involved in care is in place, but it needs improvement from the perspective of patients and families:

*Services are integrated, but we don’t have a follow-up document-for example, a flowchart that allows patients to know where to start when they access the center-although there is communication from one service to another.* (FG2, HCP7)

Ignoring these communication difficulties hinders self-care management:

*As a nurse, I notice three essential elements that are neglected: hygiene-which colleagues have already mentioned-nutrition, and another important element, communication. The person who best knows their health status is the patient. Sometimes negligence in communication between family members and nurses makes treatment more difficult. When the patient can speak, we educate them to report how they feel and how the previous day went.* (FG1, HCP9)

*The center lacks a guidance document. What we do is provide guidance based on the type of stroke each person has.* (FG3, HCP4)

Both health professionals and families stated that resources for self-care are limited by families’ socioeconomic conditions and by the broader national context:

*We have limited resources, but with what we have, we support our stroke survivors, for example, with strict medication adherence, physical exercises at home, and a set of exercises here at the center-from the exercise bike and treadmill to others needed for the patient’s condition.* (FG2, HCP6)

*The major challenge in working with stroke survivors is that they often arrive with poor hygiene due to motor and functional impairments and families’ neglect, since they do not always support their relatives in self-care and personal hygiene.* (FG3, HCP3)

*It has not been easy, given the distance between our home and the center. The transportation we have has helped. This is a health matter, and health is a priority* […] *we end up overlooking the difficulties.* (FC6)

*I had few resources, and as a college student, it was tough to handle my father’s health situation-we had to use whatever resources were within our reach.* (FC4)

*The difficulties are financial. The cost of living is high, yet a special diet and medications are needed. As a family, we try to contribute to cover these needs.* (FC9)

The lack of resources is partly offset by families’ efforts to ensure care, despite challenges such as the absence of a formal community caregiver support network, delays in care and in access to rehabilitation, and the need to manage limited family budgets to obtain necessary medications; skin-protection products (ointments, creams, lotions); and assistive devices such as wheelchairs and forearm crutches.

Regarding the category Training stroke survivors and their families for self-care management, professionals focused on implementing interventions they viewed as promoting self care, such as provision of information, health education, symptom and complication management, prevention of self-neglect, caregiver training, and support for therapeutic regimen management:

*We guide the family on the need for care and self-care for the patient-for example, informing them about the need for moderate physical exercise, attention to diet and stressors, and monitoring underlying conditions. The nursing team should guide the patient and family on the best ways to position, lie down, and move the body to prevent other problems.* (FG3, HCP8)

*Stroke results in physical, motor, and cognitive changes. Before initiating treatment, the patient undergoes a multidisciplinary assessment; in these sessions, the patient and family receive guidance on care related to health-promoting habits and lifestyle, especially regarding post-stroke sequelae. We provide guidance on gait, self-care in activities of daily living, safe bathroom use, personal hygiene, and physical activity. For those with limited mobility who are receiving outpatient treatment, we ask the family, whenever possible, to provide sun exposure to help prevent pressure ulcers and other complications.* (FG1, HCP3)

Professionals were aware that the prognosis is guarded and that, in most situations, survivors are left with permanent functional deficits. In a resource-constrained context, this leads them to prioritize training for the survivor-family dyad-and fostering accountability-for care and its continuity, as illustrated in the following verbatim excerpts:

*The greatest challenge in treating stroke survivors is reintegrating them into society, helping them return to activities of daily living* […] *we have the responsibility to educate the family who will provide home care. Our mission here is not only to treat the patient but also to return them to society.* (FG1, HCP5)

*Our focus has been to promote self-care and to train the family to care for their relative* […] *our intervention first seeks to determine the cause of the stroke and the antecedents, considering the factors reported. We teach patients how to manage self-care-related stressors and how to handle everyday conflicts. The family’s role is essential,* […]*; changing the daily habits of the patient and the family is essential to prevent recurrence.* (FG2, HCP4)

*Many patients do not adhere to their medication regimen. We have delivered educational sessions on the importance of strict adherence to therapy and on motivation for self directed recovery.* (FG1, HCP12)

This view is not shared by family members, who report needing ongoing support from health services and do not envision being without such support in the near future ort.

Regarding the last category, Training family caregivers to promote self-care, both participant groups expressed this need.

Family caregivers reported that:

*It has truly been a very challenging experience. At first, it was tough: his body was almost completely paralyzed. He had to use a wheelchair and be constantly assisted to go to the bathroom, eat, and even take his medications.* (FC1)

*Well* […] *it has been quite a difficult experience because this is the first time we are dealing with this situation. My father had a stroke on March 24, 2024. Since then, it has been about seven to eight months. In the beginning, it was very hard because his body was practically paralyzed, and to get around, he needed a wheelchair and the family’s help for all basic needs* […]. *The biggest challenge was that it was a new situation, and I had to take care of his basic needs.* (FC5)

For this participant, skills training is necessary, but he notes that:

*The center needs to be expanded* […] *motivate staff more so they can truly help* […] *be able to train us and better guide us so we can provide care at home.* (HCP4)

In light of these and other difficulties, professionals focused their attention on skills training:

*Our focus has been to promote self-care and to train the family to care for their relative.* (FG1, HCP4)

*The family needs to learn to help with hygiene care, prevention of pressure ulcers, how to lift these individuals, how to administer medications* […] *but they also need to be motivated so they do not stop providing all the necessary care.* (FG3, HCP2)

Skills training was accompanied by a concern with promoting independence:

*We conduct support sessions for patients and families here at the center. We explain how he must care for himself at home-for example, ensuring access to the bathroom, perineal care, and making the effort to adhere to the medication regimen-and gradually resume what he used to do without family assistance to regain independence.* (FG3, HCP7)

Professionals less explored the issue of transitioning into the caregiver role, but it emerged in the analysis of family-caregiver interviews as a clear concern with caregiver burden, changes in their own health, social roles, and quality of life.

## DISCUSSION

From the analysis of interviews with 9 family caregivers and FGs with 27 health professionals at a rehabilitation center in Huambo, Angola, on promoting self-care after stroke, three categories emerged: Planning and continuity of individualized rehabilitation programs; Training stroke survivors for self-care management; and Training family caregivers to promote self-care and transition into the caregiver role.

Regarding Planning and continuity of individualized rehabilitation programs, our results reinforce international recommendations to involve both survivors and families in preparing for the return home and in post-discharge continuity of care, through an effective partnership to ensure a smooth transition into the community^(19,20)^.

Continuity of care depends on the resources available to survivors and their families within the community. Participants’ accounts highlight difficulties associated with scarce-or even absent-resources and assistive products for stroke survivors’ self-care. This situation underscores the need for health professionals to rigorously plan individualized rehabilitation programs to identify needs and mobilize community resources to support these families^(3,5)^.

The results call for strengthening psychoeducational strategies that enable survivors and families to achieve independence in self-care and adherence to both pharmacological and nonpharmacological therapeutic regimens^(1-3,6,21-23)^. Behavior change is needed to adhere to the prescribed diet, practice proper positioning and mobilization, perform safe transfers, and use adaptive strategies for activities of daily living^(1,2)^. It is also necessary to ensure that survivors maintain family roles and social participation and to prevent self-neglect^(21)^.

Findings show that health professionals and families often differ in their views of the level of support and time needed for continuity of care after hospital discharge. Care should be sustained over time, with health professionals ensuring implementation of interventions and follow-up. This is a new situation for many families, who may not fully understand the condition and often do not realize that sequelae can be lifelong, reinforcing the importance of training and professional support to avoid adverse outcomes^(1,11,13)^.

A study conducted in Italy to test the effectiveness of a self-management intervention for stroke survivors, called LAY (Look After Yourself), concluded that the intervention enables survivors to develop self-management skills and facilitates the transition from hospital to community^(24,25)^. The intervention includes motivational strategies and group sessions to encourage sharing of experiences, social comparison, and vicarious (observational) learning; in this approach, individuals learn by observing others’ behavior. It also offers individual sessions focused on setting feasible action plans and teaching tailored strategies for self-care and fall prevention-events prevalent in this population^(24)^ that warrant a tailored approach by health care teams^(26)^.

The literature consistently indicates that, beyond enabling survivors and caregivers for self care management, it is important to support the family’s transition into the informal caregiver role. Accordingly, health professionals should prioritize the psychological well-being and self-care needs of caregivers of people with post-stroke sequelae, ensuring psychological and emotional support, training to support their relative without replacing them, and assistance with task management so they can have periods of respite and time to maintain social relationships^(1-3,5,27)^.

It is essential to provide comprehensive support, including psychological counseling and skills training, to strengthen caregivers’ ability to provide care^(23)^. In addition, governments and health institutions should offer family-centered support to ensure quality of life for both survivors and their caregivers^(24,25)^.

A qualitative systematic review showed that training family caregivers improves care delivery to stroke survivors and contributes to promoting survivors’ self-care^(27)^. The findings of this qualitative study do not support an association between support and training for family caregivers and the promotion of caregivers’ own self-care. However, analysis of the family-caregiver interviews indicated that supporting self-care at home has been fundamental to survivors’ functional recovery and the resumption of independence in self-care.

### Study limitations

The limitations of this study are intrinsically linked to the choice of an interpretivist design conducted in a specific context, which constrains the transferability of the findings. In addition, the use of semi-structured interviews offers flexibility and enhances the richness and depth of the results, while increasing the challenge of conducting interviews and minimizing response bias.

Triangulating data sources yielded a broader understanding of the phenomenon, increasing the rigor, depth, breadth, and transferability of the findings. Data analysis involved experts in the topic under study, whose input was crucial to coding and categorization. In turn, the analysis results were returned to participants to validate the principal investigator’s interpretations.

### Contributions to the field

This study analyzed health professionals’ and caregivers’ perceptions of promoting self-care after stroke, revealing difficulties and opportunities for improvement and informing the design of an intervention to promote survivors’ self-care and caregiver training. Pioneering in Africa, particularly in Angola, the study contributes to understanding how context can shape care delivery and affect the progression of functional ability after a stroke event.

## FINAL CONSIDERATIONS

The categories and subcategories that emerged from the analysis of interviews with family caregivers and of focus groups with health care professionals, conducted in Huambo, clarify caregivers’ and health care professionals’ perceptions of how self-care is promoted among people who have experienced a stroke. Central concerns are: planning and continuity of individualized rehabilitation programs to ensure ongoing support, communication, and access to resources; training stroke survivors for self-care management through information and education, prevention of self-neglect, and support for therapeutic regimen management; and training family caregivers to promote self-care and transition into the caregiver role, with an emphasis on skills training to foster independence in self-care among people with stroke.

## Data Availability

The research data are available only upon request.

## References

[1] Mou H, Lam SKK, Chien WT. (2023). The effects of a family-focused dyadic psychoeducational intervention for stroke survivors and their family caregivers: a randomised controlled trial. Int J Nurs Stud.

[2] Mou H, Lam SKK, Chien WT. (2022). Effects of a family-focused dyadic psychoeducational intervention for stroke survivors and their family caregivers: a pilot study. BMC Nurs.

[3] Tsang WN, Lee JJ, Yang SC, Poon JCY, Lau EYY. (2024). Stroke caregivers’ perception on instant messaging application use for psychological intervention: a qualitative study. Psychol Health Med.

[4] Riegel B, Dunbar SB, Fitzsimons D, Freedland KE, Lee CS, Middleton S (2021). Self-care research: where are we now? where are we going?. Int J Nurs Stud.

[5] Chen T, Zhang B, Deng Y, Fan J-C, Zhang L, Song F. (2019). Long-term unmet needs after stroke: systematic review of evidence from survey studies. BMJ Open.

[6] Fianu GK. (2023). Experiences of family caregivers of patients with stroke in the Cape Coast Metropolis: a descriptive qualitative design.

[7] Prasetyowati CD, Firmanda GI. (2024). Empowering post-stroke patients to improve self-care and prevent recurrent stroke using stroke empowerment education. J Keperawatan Komprehensif.

[8] Rasyid A, Pemila U, Aisah S, Harris S, Wiyarta E, Fisher M. (2023). Exploring the self-efficacy and self-care-based stroke care model for risk factor modification in mild-to-moderate stroke patients. Front Neurol.

[9] Choo PY, Shaik MA, Tan-Ho G, Lee J, Ho AHY. (2023). Living losses in stroke caregiving: a qualitative systematic review of systematic reviews on psycho-socio-emotional challenges and coping mechanisms. Int J Stroke.

[10] Bhagavathy MG, Anniyappa S, Thankappan R, Bharathi B. (2022). Lived experiences of stroke survivors in India: a phenomenological study. BNJ.

[11] Costa FM, Canto DF, Felipe LT, Rosset I, Paskulin LMG. (2024). Educational interventions for training caregivers of stroke survivors: a scoping review. Texto Contexto Enferm.

[12] Stockbridge MD, Bunker LD, Hillis AE. (2022). Reversing the ruin: rehabilitation, recovery, and restoration after stroke. Curr Neurol Neurosci Rep.

[13] Rojas-Ocaña MJ, Araujo-Hernández M, Romero-Castillo R, García Navarro EB. (2021). Educational interventions by nurses in caregivers with their elderly patients at home. Prim Health Care Res Dev.

[14] de Oliveira ESF, Baixinho CL, Presado M. (2019). Qualitative research in health: a reflective approach. Rev Bras Enferm.

[15] Krueger RA, Casey MA. (2014). Focus Groups: a practical guide for applied research.

[16] Renz SM, Carrington JM, Badger TA. (2018). Two strategies for qualitative content analysis: an intramethod approach to triangulation. Qual Health Res.

[17] Denzin NK, Lincoln YS. (2018). The Sage handbook of qualitative research.

[18] Tong A, Sainsbury P, Craig J. (2007). Consolidated criteria for reporting qualitative research (COREQ): a 32-item checklist for interviews and focus groups. Int J Qual Health Care.

[19] Mountain A, Lindsay MP, Teasell R, Salbach NM, Jong A, Foley N (2020). Canadian stroke best practice recommendations: rehabilitation, recovery, and community participation following stroke. Part two: transitions and community participation following stroke. Int J Stroke.

[20] Johansson U, Hellman T, Öst Nilsson A, Eriksson G. (2021). The ReWork-Stroke rehabilitation programme described by use of the TIDieR checklist. Scand J Occup Ther.

[21] Kuo NY, Lin YH, Chen HM. (2021). Continuity of care and self-management among patients with stroke: a cross-sectional study. Healthcare.

[22] Chi N-F, Huang Y-C, Chiu H-Y, Chang H-J, Huang H-C. (2020). Systematic review and meta-analysis of home-based rehabilitation on improving physical function among home-dwelling patients with a stroke. Arch Phys Med Rehabil.

[23] Nungulo VN, Gonçalves M, Henriques MA, Baixinho CL. (2025). Effectiveness of psychoeducational intervention in promoting post-stroke self-care: a systematic literature review. Front Rehabil Sci.

[24] Fugazzaro S, Denti M, Accogli MA, Costi S, Pagliacci D, Calugi S (2021). Self-management in stroke survivors: development and implementation of the look after yourself (LAY) intervention. IJERPH.

[25] Messina R, Dallolio L, Fugazzaro S, Rucci P, Iommi M, Bardelli R (2020). The Look After Yourself (LAY) intervention to improve self-management in stroke survivors: results from a quasi-experimental study. Patient Educ Couns.

[26] Baixinho CL, Dixe MA. (2017). Team practices in fall prevention in institutionalized elderly people: scale design and validation. Texto Contexto Enferm.

[27] Chen H, Makhdzir N, Loh WC, Wang L. (2025). Quality of life of family caregivers of stroke patients: a systematic review of qualitative research. Pak J Med Sci.

