# Supplementary material for: Self-care in stroke rehabilitation: a qualitative study
Source: Rev Bras Enferm. 2026 Jul 17;79:e20250332. doi: 10.1590/0034-7167-2025-0332 (PMC13379239; doi:10.1590/0034-7167-2025-0332)
Supplement: Translation

# Translation: O autocuidado na reabilitação do acidente vascular cerebral: uma pesquisa qualitativa

**DOI:** 10.1590/0034-7167-2025-0332pt

**Authors:** Victor Nhime Nungulo, Mauer Gonçalves, Maria Adriana Henriques, Cristina Lavareda Baixinho

**Affiliations:** I Universidade José Eduardo dos Santos. Huambo, Angola; II Universidade de Lisboa. Lisboa, Portugal; III Universidade Agostinho Neto. Luanda, Angola; IV Center for Innovative Care and Health Technology. Leiria, Portugal

**Keywords:** Autocuidado, Cuidadores, Pessoal de Saúde, Pesquisa Qualitativa, Reabilitação do Acidente Vascular Cerebral

## Abstract

**Objetivos:**

analisar as percepções dos cuidadores e profissionais sobre as intervenções que promovem o autocuidado na pessoa sobrevivente de Acidente Vascular Cerebral.

**Métodos:**

estudo qualitativo inserido no paradigma interpretativista. Realizaram-se nove entrevistas com familiares cuidadores e três *focus groups* com profissionais de saúde. Colheram-se os dados em Angola, entre dezembro de 2024 e janeiro de 2025, com gravação e transcrição para análise de conteúdo com WebQDA^®^.

**Resultados:**

são preocupações centrais o planeamento e continuidade dos programas individuais de reabilitação, para garantir apoio, comunicação e recursos; capacitação para a autogestão do autocuidado em termos de informação, educação, prevenção da negligência e ajuda na gestão terapêutica; e capacitação do familiar cuidador para promoção do autocuidado e transição para o papel de cuidador.

**Considerações Finais:**

os resultados permitem compreender a percepção dos familiares e profissionais de saúde sobre a promoção do autocuidado em sobreviventes de Acidente Vascular Cerebral, gerando recomendações para a clínica e investigação.

## INTRODUÇÃO

O acidente vascular cerebral (AVC) é uma das principais causas de incapacidade e morte, prejudicando a capacidade funcional e a qualidade de vida não só dos doentes, mas também dos cuidadores e familiares^(1-3)^. Após o AVC, a pessoa fica com incapacidade, temporária ou permanente, que afeta a capacidade de autocuidado, tornando-se dependente de um cuidador ou de um profissional de saúde para a realizar as atividades de vida diária. As alterações no autocuidado são uma preocupação, porque repercutem na evolução da funcionalidade nos meses seguintes ao surgimento da doença^(4,5)^.

Uma revisão sistemática mostrou que essas pessoas apresentam múltiplas necessidades decorrentes de comprometimentos físicos, incapacidade funcional, labilidade emocional, perda cognitiva, fadiga e dor crónica, bem como mudanças na participação social e familiar causadas essencialmente pelas limitações na mobilidade e na comunicação^(1-5)^. Acrescem ainda necessidades não satisfeitas, relacionadas com as dificuldades de acesso aos cuidados de saúde e à continuidade de cuidados entre o hospital, as unidades de reabilitação e/ou o domicílio^(5)^.

As sequelas secundárias ao AVC, associadas às dificuldades dos sistemas de saúde em garantir a continuidade de cuidados e o suporte de longa duração a essas pessoas, limitam o autocuidado e a adoção de comportamentos que asseguram um estilo de vida saudável, o equilíbrio emocional e a participação na vida social^(4-6)^.

Os estudos internacionais observam que várias são as intervenções que podem promover o autocuidado a esses doentes, permitindo-lhes o envolvimento em atividades da vida diária e outras atividades para preservar o bem-estar biopsicossocial^(7,8)^. Salienta-se o crescente interesse de pesquisadores pelas intervenções psicoeducativas, as quais têm demonstrado efetividade na capacitação dessas pessoas e dos seus cuidadores para serem independentes no autocuidado e na gestão do seu processo de saúde-doença^(1-3,8,9)^. Tais ações favorecem a reintegração dessas pessoas na comunidade^(9,10)^ e desenvolvem a competência dos cuidadores para auxiliar, sem substituir, o autocuidado, ampliando a literacia em saúde e a capacidade de articular recursos comunitários para atender às necessidades de doentes e famílias^(5,11)^. Além disso, reduzem o impacto económico sobre os sistemas de saúde, associado ao tratamento das sequelas, complicações e reinternações^(12)^.

As principais intervenções psicoeducativas implementadas com pessoas com AVC e suas famílias são o fornecimento de informação; a educação de cuidadores, com o treinamento para execução de cuidados diários e reabilitação física; o apoio psicológico com aconselhamento e suporte, promovendo respostas emocionais positivas ao estresse diário; o apoio emocional, por meio de atividades de apoio em grupo, escuta ativa ou psicoterapia; a ajuda na identificação de prioridades e resolução de problemas; a regulação comportamental e emocional, que modifica os comportamentos para melhorar resultados de saúde; e o apoio social, incluindo a identificação de serviços de saúde e da comunidade e o modo de acessá-los, bem como estratégias para obter ajuda da família e dos amigos^(1,2,13)^.

Os estudos revelam que essas intervenções são mais eficazes se forem implementadas durante a internação como parte do planeamento da alta^(7,13)^. A assistência no domicílio é essencial para atender às necessidades do doente e reduzir a sobrecarga da família na transição para o papel de cuidador; por isso, seu planeamento deve começar ainda na internação, antes da alta^(13)^. A literatura indica que a implementação de programas psicoeducativos é mais eficaz quando têm duração mínima de três meses e inclui a capacitação de cuidadores^(1,2,13)^. Os autores recomendam que o enfermeiro lidere esses programas, conduza sua implementação e avalie sua efetividade^(13)^.

A escassez de estudos sobre esse tema em África, muito concretamente em Angola, justifica o desenvolvimento deste estudo.

## OBJETIVOS

Analisar as percepções dos familiares cuidadores e profissionais de saúde sobre as intervenções que promovem o autocuidado na pessoa sobrevivente de AVC.

## MÉTODOS

### Aspectos éticos

O estudo foi conduzido de acordo com as diretrizes de ética nacionais e internacionais e aprovado pela comissão de ética do Centro de Medicina e de Reabilitação Física Princesa Diana, Huambo, Angola. Os familiares e profissionais assinaram o Termo de Consentimento Livre e Esclarecido, autorizando a participação no estudo e as gravações. Eles foram informados de que poderiam desistir do estudo em qualquer momento. Foram respeitados os aspetos éticos relativos à pseudonomização e confidencialidade dos achados. O conteúdo dos *focus groups* (FG) e entrevistas (E) foi transcrito e, depois, armazenado no software de análise qualitativa de dados, que está alojado em nuvem com acesso restrito mediante utilizador e *password*. Para preservar anonimato e confidencialidade, os achados foram apresentados com codificação alfanumérica: grupos focais como FG1, FG2, FG3; profissionais de saúde como PS1, PS2…, PSn; e familiares como F1, F2…, Fn.

### Tipo de estudo

A questão de pesquisa foi: “Que percepções têm os familiares cuidadores e profissionais de saúde sobre as intervenções que promovem o autocuidado na pessoa sobrevivente de AVC?”. Para respondê la, optou-se por um estudo inserido no paradigma interpretativista. Os estudos qualitativos possibilitam aprofundar fenómenos complexos e, concomitantemente, compreender as experiências num contexto específico e sob a perspetiva dos participantes sobre a solução para questões intricadas da realidade^(14)^. O facto de a revisão da literatura ter possibilitado verificar a escassez de estudos sobre o tema em Angola reforça a escolha dos métodos: entrevistas semiestruturadas e *focus group* ^(15-17)^.

O FG favorece e incentiva a compreensão, descrição e análise da realidade por meio da dinâmica das relações sociais; aborda o universo de significados, motivos, aspirações, crenças, percepções, valores, atitudes, opiniões e interpretações sobre como as pessoas, vivem, sentem e pensam; e permite a interação do grupo com base no papel ativo do investigador na dinamização da discussão para efeito de recolha de dados^(15)^.

Os FGs foram planeados de acordo com as orientações de Krueger e Casey, cumprindo um protocolo que incluiu as etapas de planeamento, preparação, moderação e análise dos dados e divulgação dos resultados^(15)^.

Primeiramente, efetuaram-se as entrevistas com os cuidadores; em seguida, os FGs com os profissionais de saúde. Por último, fez-se a triangulação dos dados, combinando aqueles de fontes diferentes^(16)^. Esse procedimento levou a uma maior compreensão da realidade e possibilitou desvelar aspetos diferentes do fenómeno investigado^(17)^.

Para a elaboração do artigo, seguiram-se as recomendações da COREQ (*COnsolidated criteria for REporting Qualitative research*)^(18)^.

### Cenário do estudo

Os participantes deste estudo foram os familiares cuidadores e os profissionais de saúde, os quais prestavam cuidados a pessoas que haviam sofrido acidente vascular cerebral e estavam em processo de reabilitação no Centro de Medicina e Reabilitação Física Princesa Diana-Huambo, Angola. O centro tem a particularidade de receber doentes que estão no domicílio e recorrem ao serviço para continuar o seu programa de reabilitação, para aqueles casos com e/ou sem internação. Portanto, a continuidade de cuidados no domicílio é de responsabilidade da família.

Foram realizadas nove entrevistas com familiares cuidadores e três *focus groups* com profissionais de saúde, apoiando-se num guião semiestruturado, ajustado a cada grupo de participantes. No FG, optou-se por uma amostra homogénea para focalizar a discussão no tema e aprofundar os resultados. Os participantes só participaram em um dos estudos, de acordo com os critérios pré-definidos.

### Fonte de dados

Os dados foram colhidos entre dezembro de 2024 e janeiro de 2025, no Centro de Medicina e de Reabilitação Física Princesa Diana, Huambo, Angola.

Após autorização da direção do centro, foram identificados os potenciais participantes. O investigador responsável por este estudo fez um primeiro contacto com os profissionais e familiares identificados, para verificar se preenchiam os critérios de elegibilidade.

Foi elaborado um guião para a entrevista semiestruturada. Manteve-se a questão central: “Como promove o autocuidado na pessoa sobrevivente de AVC?”. Com base nela, foi possível desdobrá-la em questões mais específicas.

Os FGs foram conduzidos pelo mesmo moderador, que também realizou as entrevistas; ele foi apoiado por um comoderador, o qual fez apontamentos sobre a discussão^(15)^. Os FGs foram presenciais, e o consentimento dos participantes foi obtido antes das sessões, gravadas em áudio e vídeo. O tempo médio de cada FG foi de 90 minutos; e o das entrevistas, 45 minutos.

### Análise de dados

Após a transcrição das entrevistas e dos FGs, a análise de dados seguiu as recomendações para a análise dos achados em pesquisa qualitativa^(17)^. Codificaram-se os dados e atribuíram-se as respetivas categorias, tendo em conta as questões elencadas no guião de entrevista, assim como aquelas que foram surgindo ao longo das entrevistas e dos FGs. Os achados foram armazenados/recuperados depois da transcrição. Os dados textuais similares foram agrupados na mesma categoria, proporcionando, assim, uma análise organizada e sistematizada dos dados. A análise foi feita com recurso a um software de análise qualitativa (WebQDA^®^).

A codificação foi efetuada pelo investigador encarregado da transcrição e foi validada pelos outros membros da equipa de trabalho. Durante a categorização, foram tidos em conta a representatividade, a saturação dos dados, a homogeneidade, a pertinência e a exaustividade.

As entrevistas e os FGs foram analisados individualmente. Posteriormente foi feita a triangulação dos resultados dos dois estudos, comparando os códigos para identificar as semelhanças e diferenças entre as categorias e respetivas subcategorias^(16,17)^.

## RESULTADOS

Foram realizados três FGs com profissionais de saúde (FG1, n = 12; FG2, n = 7; e FG3, n = 8) e nove entrevistas com familiares cuidadores (n = 9). Dos nove familiares participantes deste estudo, seis são homens e três são mulheres, tendo a média de idade de 34,5 (±9,55), com o mínimo de 28 e o máximo de 54 anos.

Os 27 profissionais de saúde enquadraram-se nas seguintes categorias profissionais: enfermeiro (n = 12), fisioterapeuta (n = 10), assistente social (n = 2), psicólogo (n = 1), médico (n = 1), cardiopneumologista (n = 1). Foram 18 mulheres e 9 homens, com idades compreendidas entre 29 e 52 anos [39,1(±6,39)]. O tempo médio de exercício profissional é de 10 anos.

Na análise dos FGs e das entrevistas, emergiram 208 unidades de registo, distribuídas nas seguintes categorias: 1) Planeamento e continuidade dos programas individuais de reabilitação, tendo as subcategorias: apoio, comunicação e recursos para o autocuidado; 2) Capacitar a pessoa/família para a autogestão do autocuidado, com as subcategorias: intervenções dos profissionais promotoras do autocuidado (informação para o autocuidado, educação, prevenção da negligência, capacitação do cuidador e ajuda na gestão terapêutica) e responsabilização; 3) Capacitar o familiar cuidador, com as subcategorias: treino de habilidades, suporte para a promoção da independência e transição para o papel de cuidador (Quadro 1).

**Quadro 1:** Representação das categorias e subcategorias que emergiram na análise dos achados, Huambo, Angola, 2025

Categorias	Subcategorias
Planeamento e continuidade dos programas individuais de reabilitação	Apoio Comunicação Recursos para o autocuidado
Capacitar a pessoa/família para a autogestão do autocuidado	Intervenções dos profissionais promotoras do autocuidado Responsabilização
Capacitar o familiar cuidador	Treino de habilidades Suporte para a promoção da independência Transição para o papel de cuidador

No que diz respeito à categoria Planeamento e continuidade dos programas individuais de reabilitação, os participantes destacaram a importância do apoio à díade doente/família para assegurar a continuidade de cuidados em casa.

O discurso dos familiares centrou-se nas necessidades de apoio, porque, após um evento de AVC:

*O maior desafio é ter sido uma situação nova e ter de cuidar das necessidades básicas dele.* (F5)

Constataram que, no hospital, não há tempo/oportunidade para receberem explicações sobre o que fazer em relação às limitações dos pacientes relacionadas com a hemiparesia, hemiplegia, dificuldades de mobilização e de comunicação. Por isso, o apoio dado pelos profissionais, no centro de reabilitação, é importante não só para os ajudar a compreender as sequelas e implicações da doença no autocuidado, mas também aprender os procedimentos a fim de garantir a continuidade de cuidados em casa, dado que esses doentes deslocam-se ao centro para a reabilitação, mas não ficam internados. Um familiar explica essa situação:

[...] *na primeira consulta, dão sempre uma orientação de como cuidar e também ensinar a ela como se autocuidar. Explicaram-me para não estar ansiosa, cuidar dela nos banhos e na alimentação e ensinar-lhe a fazer a higiene pessoal* [...] *para que possa ficar capaz de o fazer sozinha.* (F8)

Os profissionais do centro de reabilitação estavam cientes da não preparação dos doentes e famílias para essa nova realidade de dependência: são adultos que, até então, eram independentes para o autocuidado:

*O tratamento de pacientes com sequelas de AVC é desafiador, porque a problemática começa desde o seio familiar, desde a situação económica e dificuldades de transporte do paciente da sua casa para o centro. Um dos grandes desafios, muitas vezes, é o facto de que o próprio paciente e a família não aceitam a condição da doença,* [...] *um dos grandes problemas é mesmo a aceitação e os cuidados a terem com o paciente sobrevivente de AVC, principalmente os familiares, para darem sequência aos cuidados em casa.* (FG1, PS6)

A falta de preparação das famílias para lidar com a situação, as alterações funcionais dos doentes, as dificuldades de acesso ao centro de reabilitação por motivos de transporte ou até de não consciencialização da necessidade de manter um programa de reabilitação levam a que os profissionais optem por visitas domiciliárias para apoiar a díade:

*Fizemos, muitas vezes, visitas domiciliares para ver se o paciente tem condições de realizar as suas atividades de vida diária e lhe ensinar a se autocuidar* [...] *o apoio psicológico também é muito importante.* (FG2, PS5)

[...] *em relação ao acompanhamento dos nossos pacientes no domicílio, é preciso primeiro ver em que condições vive o doente e orientar as adaptações na sua residência. Por exemplo, para quem usa cadeiras de rodas, muitas vezes a casa não está preparada para o efeito, e isso pode dificultar. Conhecer essas condições evita que o paciente sofra um acidente no domicílio. Tal como a minha colega se referiu anteriormente, apoiar os cuidadores é também importante, porque eles pensam que o tratamento é feito em poucos dias* [...] *não! O tratamento do AVC envolve seguimento contínuo, e conseguir o envolvimento da família é fundamental. A família tem de saber que o seu paciente está com este problema, mas não é o fim da vida. E ensinar ao paciente a aceitar que está nesta condição, isso ajuda ao paciente para se motivar para a recuperação e voltar a realizar as suas atividades de vida diária, apesar de, algumas vezes, deixar sequelas irreversíveis.* (FG2, PS7)

Todos os entrevistados concordaram que a comunicação é importante e central para a capacitação e continuidade de cuidados, mas relataram dificuldades na comunicação (verbal e escrita) tanto entre equipes quanto no interior das equipes e na interlocução com doentes e familiares.

A comunicação entre serviços e profissionais envolvidos no cuidado é garantida, porém necessita de aprimoramento na ótica do doente e da família:

*Os serviços estão integrados, mas não temos um documento para o seguimento - por exemplo, um fluxograma que permita que os pacientes saibam onde começar quando acedem ao centro - mas existe comunicação entre um serviço e outro.* (FG2, P7)

Negligenciar essas dificuldades comunicativas prejudica a autogestão da doença:

*Enquanto enfermeiro, noto três elementos muito importantes que são negligenciados: a questão da higiene, que já foi dita aqui pelos colegas; a alimentação; e outro elemento importante é a comunicação. Quem melhor conhece a sua condição de saúde é o próprio doente. Às vezes, a negligência na comunicação entre os familiares e os enfermeiros dificulta o tratamento. Quando o paciente é capaz de falar, educamo-lo para que nos informe sobre como se sente e como passou o dia anterior.* (FG1, P9)

*O centro não tem um documento orientador. O que fazemos é dar orientações com base no tipo de AVC que cada um tem.* (FG3, P4)

Tantos os profissionais de saúde como as famílias expressaram que os recursos para o autocuidado são limitados pelas condições socioeconómicas das famílias e do próprio país:

*Temos poucos recursos, mas, com base no que temos, apoiamos os nossos pacientes sobreviventes de AVC, como, por exemplo, no cumprimento escrupuloso da medicação, realização de exercícios físicos em casa e uma bateria de exercícios aqui no centro: desde a bicicleta e a esteira até outros necessários à situação do doente.* (FG2, P6)

*O grande desafio em trabalhar com os pacientes sobreviventes de AVC é o facto de que muitas vezes chegam com pouca higiene, por causa das debilidades motoras e funcionais e do desleixo das famílias, que nem sempre apoiam os seus pacientes no autocuidado e no cuidado da higiene pessoal.* (FG3, PS3)

*Não tem sido fácil, dada a distância entre a nossa residência e o centro. O meio de transporte que temos tem-nos facilitado. Trata-se de uma questão de saúde, e a saúde é prioridade* [...] *acabamos esquecendo as dificuldades.* (F6)

*Tive poucos recursos e, enquanto estudante da faculdade, foi mesmo difícil para lidar com a situação de saúde do meu pai: tínhamos de usar os recursos que estavam ao nosso alcance.* (F4)

*As dificuldades são do ponto de vista financeiro. O custo de vida está alto, mas precisa de uma dieta própria e aquisição de medicamentos. Enquanto família, procuramos fazer contribuições para suprir essas necessidades.* (F9)

A falta de recursos é suportada pelo esforço das famílias em garantir cuidados, diante das dificuldades associadas à ausência de uma rede de suporte comunitária de cuidadores formais, atraso no atendimento e acesso à reabilitação, gestão dos orçamentos familiares limitados para assegurar a aquisição de alguma medicação necessária, de pomadas/cremes/loções para a proteção da pele e de produtos de apoio como cadeira de rodas e canadianas, entre outros.

Em relação à categoria Capacitar a pessoa/família para a autogestão do autocuidado, houve uma preocupação dos profissionais em implementar intervenções que consideraram promotoras do autocuidado, tais como transmissão de informação, educação para a saúde, gestão de sintomas e de complicações, prevenção da negligência, capacitação do cuidador e ajuda na gestão terapêutica:

*Orientamos a família sobre a necessidade de cuidados e autocuidado com o paciente: por exemplo, informar sobre a necessidade de fazer exercícios físicos moderados, cuidar da alimentação e dos fatores estressores e prestar atenção às patologias de base. A equipe de enfermagem tem de orientar o paciente e a família sobre as melhores formas de se posicionar, deitar e até mesmo sobre a mobilidade do corpo, para que não venha a ter outros problemas.* (FG3, P8)

*O AVC é uma doença que gera alterações físicas, motoras e cognitivas. Antes do tratamento, o paciente é submetido a uma observação multidisciplinar; e, nessas sessões, é orientado* [ele e sua família] *sobre os cuidados em relação a hábitos e estilo de vida que promovam a saúde, levando em conta, principalmente, as sequelas do pós-AVC. Orientamos sobre a marcha, autocuidado nas atividades do dia a dia, modo de usar o banheiro, higiene pessoal, atividade física. E para aqueles que tem pouca mobilidade e estão a fazer o tratamento ambulatório, pedimos à família para, sempre que possível, fazer um banho de sol a fim de evitar as escaras de decúbito e outras complicações.* (FG1, P3)

Os profissionais estavam conscientes de que o prognostico é reservado e que, na maioria das situações, a pessoa fica com défices funcionais permanentes. Por isso, um contexto de parcos recursos leva-os a ter uma preocupação clara em capacitar e responsabilizar a díade pelos cuidados e pela sua continuidade, como fica expresso nestes excertos verbatim:

*O maior desafio em tratar pacientes sobreviventes de AVC é reintegrá-los na sociedade, fazer com que voltem a fazer as suas atividades de vida diária* [...] *temos a responsabilidade de educar a família que vai cuidar do paciente em contexto domiciliar. A nossa missão aqui não é apenas tratar do paciente, mas também devolvê-lo à sociedade.* (FG1, P5)

*O nosso foco tem sido a promoção do autocuidado e capacitação da família para o cuidado do seu doente* [...] *a nossa intervenção tem sido no sentido de primeiro saber a causa do AVC e os antecedentes, tendo em conta os fatores por ele anunciado. Vamos educar os pacientes com vista à gestão dos fatores estressores para o seu autocuidado e ensiná-los a gerir os conflitos da vida diária. O papel da família é fundamental,* [...]*, mudar os hábitos diários do paciente e da família é fundamental para não voltar a ter recaídas.* (FG2, P4)

*Muitos pacientes não cumprem com o tratamento medicamentoso. Temos feito palestras sobre a importância do cumprimento escrupuloso da terapêutica e acerca da motivação para a autorrecuperação.* (FG1, PS12)

Essa visão não é partilhada pelos familiares que manifestam a necessidade de apoio contínuo dos cuidados de saúde e não perspetivam um futuro próximo sem esse suporte.

No que concerne à última categoria, Capacitar o familiar cuidador, ambos os grupos de participantes declararam essa necessidade.

Os familiares consideraram que:

*Realmente tem sido uma experiência bastante desafiadora. No início, foi muito difícil: ficou com o corpo quase totalmente paralisado. Teve de usar uma cadeira de roda e ser levado constantemente para ir ao banheiro, comer e até mesmo tomar os medicamentos.* (F1)

*Prontos* [...] *realmente tem sido uma experiência um tanto quanto difícil, pois que é a primeira vez que estamos a lidar com essa situação. O meu pai foi acometido com essa doença no dia 24 de março de 2024. De lá para cá, vão sensivelmente de sete a oito meses. No princípio, foi muito difícil, porque o corpo ficou praticamente todo paralisado e, para se locomover, precisou de uma cadeira de roda e auxílio da família para todas as necessidades básicas* [...]. *O maior desafio é ter sido uma situação nova e ter de cuidar das necessidades básicas dele.* (F5)

Para este participante, o treino de habilidades é necessário, mas observa que:

*O centro precisa ser ampliado* [...] *motivar mais os funcionários para que possam de facto ajudar* [...] *serem capazes de nos capacitar, de melhor nos orientar para conseguirmos cuidar em casa.* (P4)

Em face destas e outras dificuldades, os profissionais centraram a sua atenção no treino de habilidades:

*O nosso foco tem sido a promoção do autocuidado e capacitação da família para o cuidado do seu doente.* (FG1, P4)

*A família precisa de aprender a ajudar nos cuidados de higiene, na prevenção de escaras, como levantar estas pessoas, como dar a medicação* [...] *mas também precisa de ser motivada para não deixar de prestar os cuidados todos.* (FG3, P2)

O treino de habilidades foi acompanhado de uma preocupação com a promoção da independência:

*Realizamos sessões de apoio ao paciente e família aqui no centro. Explicamos sobre como ele tem de cuidar de si em casa: por exemplo, ter acesso a casa de banho, o cuidado da higiene íntima e a necessidade de fazer esforço para o cumprimento da medicação e aos poucos ir fazendo aquilo que fazia sem o apoio da família para o retorno à independência.* (FG3, P7)

Menos explorada pelos profissionais foi a questão da transição para o papel de cuidador, mas que emergiu na análise das entrevistas com os familiares cuidadores por meio de uma preocupação clara com a sobrecarga enquanto cuidador, alterações na sua saúde, papéis sociais e qualidade de vida.

## DISCUSSÃO

Da análise dos achados das entrevistas com 9 familiares cuidadores e dos FGs com 27 profissionais de saúde de um centro de reabilitação (Huambo, Angola) sobre a promoção do autocuidado após o AVC, emergiram três categorias: Planeamento e continuidade dos programas individuais de reabilitação, Capacitação da pessoa/família para a autogestão do autocuidado e Capacitação do familiar cuidador.

Quanto à categoria Planeamento e continuidade dos programas individuais, os resultados desta pesquisa reforçam as recomendações internacionais sobre a necessidade de envolver tanto o doente como a família na preparação do regresso à casa e na continuidade de cuidados pós-alta, mediante uma parceria efetiva para garantir uma boa transição para a comunidade^(19,20)^.

A continuidade de cuidados sempre depende dos recursos que as pessoas e as suas famílias têm e dispõem na comunidade. O discurso dos participantes revela as dificuldades associadas à escassez ou mesmo inexistência de recursos e produtos de apoio para o autocuidado da pessoa sobrevivente de AVC. Essa situação reforça a necessidade de um planeamento rigoroso dos programas individuais de reabilitação por parte de profissionais de saúde para identificar necessidades e recursos comunitários que possam ser mobilizados para o apoio a essas famílias^(3,5)^.

Os resultados deste estudo são um repto para a valorização do uso de estratégias psicoeducativas que permitam capacitar os doentes e as famílias para a independência no autocuidado da pessoa sobrevivente de AVC e para a adesão ao regime terapêutico, medicamentoso e não medicamentoso^(1-3,6,21-23)^. Nesse sentido, é preciso mudança comportamental para o cumprimento da dieta prescrita, posicionamentos e mobilização, bem como levante e estratégias adaptativas na execução das atividades de vida diária^(1,2)^. Acresce ainda a necessidade de garantir que esses doentes mantenham os seus papéis na família e a interação social e de prevenir comportamentos de negligência no autocuidado^(21)^.

Os achados mostram que os profissionais de saúde e as famílias não têm a mesma visão sobre a necessidade de apoio e tempo para a continuidade de cuidados após a alta hospitalar. Esses cuidados devem ser mantidos ao longo do tempo, com os profissionais de saúde a garantirem a implementação de intervenções e *follow-up* desses doentes. De salientar que esta é uma situação nova para as famílias, as quais podem não compreender a doença e, muitas vezes, não perceber que as sequelas serão para a vida, o que reforça a importância da capacitação e suporte dos profissionais para evitar desfechos desfavoráveis^(1,11,13)^.

Um estudo conduzido em Itália a fim de testar a eficácia de uma intervenção de autogestão para sobreviventes de AVC, designada de LAY (*Look After Yourself -* em tradução livre, “Cuide de Si”), concluiu que a intervenção possibilita desenvolver competências de autogestão em sobreviventes de AVC, facilitando a transição do hospital para a comunidade^(24,25)^. A intervenção inclui estratégias motivacionais e sessões de grupo para promover a partilha de experiências, a comparação social e a aprendizagem observacional (vicariante); nessa abordagem, a pessoa aprende ao observar o comportamento de outrem. Há ainda sessões individuais centradas em definir planos de ação exequíveis e em ensinar estratégias personalizadas para o autocuidado e para a prevenção de quedas - eventos prevalentes nesse grupo de doentes^(24)^, justificando uma intervenção diferenciada das equipas de saúde^(26)^.

Os resultados da investigação são consensuais sobre o fato de que, para além de capacitar as pessoas e cuidadores para a autogestão do autocuidado, importa apoiar a família na transição para o papel de cuidador informal. Nesse sentido, os profissionais de saúde devem dar prioridade ao bem estar psicológico e às necessidades de autocuidado dos cuidadores das pessoas com sequelas pós-AVC, assegurando apoio psicológico e emocional, formação para apoiar o seu familiar sem substitui-lo e apoio na gestão das tarefas por forma a terem períodos de descanso e tempo para manter as relações sociais^(1-3,5,27)^.

É essencial oferecer um apoio abrangente, incluindo aconselhamento psicológico e formação profissional, para melhorar as suas capacidades de prestação de cuidados^(23)^. Além disso, os governos e as instituições de saúde devem oferecer apoio centrado na família para garantir a qualidade de vida tanto dos doentes como dos seus cuidadores^(24,25)^.

Uma revisão sistemática qualitativa demonstrou que a capacitação do familiar cuidador melhora a prestação de cuidados às pessoas sobreviventes de AVC e contribui para a promoção do autocuidado nessas pessoas^(27)^. Os achados deste estudo qualitativo não permitem a associação entre o suporte e a capacitação do familiar cuidador e a promoção do seu autocuidado. Porém, a análise do conteúdo das entrevistas com os familiares evidenciou que apoiar o autocuidado no domicílio tem sido fundamental para a recuperação funcional dos doentes e retoma da independência no autocuidado.

### Limitações do estudo

As limitações deste estudo estão intrinsecamente associadas à escolha de uma pesquisa de natureza interpretativista realizada num contexto específico, o que limita a transferibilidade dos resultados. Acresce que o recurso a entrevistas semiestruturadas possibilita alguma flexibilidade e eleva a riqueza e a profundidade dos resultados, ao passo que aumenta a dificuldade de conduzir as entrevistas e eliminar o viés associado à resposta.

A triangulação de fontes levou a uma maior compreensão da realidade, aumentando o rigor, a profundidade, extensão e transferibilidade dos resultados. A análise dos dados envolveu a participação de experts na temática em estudo, cujas intervenções foram determinantes no processo de codificação e categorização. Por outro lado, os resultados da análise foram devolvidos aos participantes para validação das interpretações feitas pelo investigador principal.

### Contribuições para a área

Este estudo analisou as percepções de profissionais e cuidadores sobre a promoção do autocuidado após o AVC, desvelando dificuldades e oportunidades de melhoria e contribuindo para o desenho de uma intervenção destinada a promover o autocuidado dos doentes e a capacitação dos cuidadores. Pioneiro em África e particularmente em Angola, o estudo contribui para a compreensão de como o contexto pode influenciar a prestação de cuidados e interferir na evolução da funcionalidade após um evento de AVC.

## CONSIDERAÇÕES FINAIS

As categorias e subcategorias que emergiram da análise das entrevistas com familiares cuidadores e dos *focus groups* com profissionais de saúde permitem compreender a percepção dos familiares e profissionais de saúde sobre a promoção do autocuidado em pessoas sobreviventes de acidente vascular cerebral, no Huambo. São preocupações centrais: o planeamento e continuidade dos programas individuais de reabilitação, por forma a garantir apoio, comunicação e recursos para o autocuidado; a capacitação para a autogestão do autocuidado em termos de informação, educação, prevenção da negligência, capacitação do cuidador e ajuda na gestão terapêutica; e a capacitação do familiar cuidador para o desenvolvimento de habilidades a fim de assegurar a promoção da independência no autocuidado da pessoa com AVC e transição para o papel de cuidador.

## Data Availability

Os dados de pesquisa só estão disponíveis mediante solicitação.
